# The Associations Between Racially/Ethnically Stratified COVID-19 Tweets and COVID-19 Cases and Deaths: Cross-sectional Study

**DOI:** 10.2196/30371

**Published:** 2022-05-30

**Authors:** Xiaohui Liu, Bandana Kar, Francisco Alejandro Montiel Ishino, Tracy Onega, Faustine Williams

**Affiliations:** 1 National Institute on Minority Health and Health Disparities National Institutes of Health Bethesda, MD United States; 2 Huntsman Cancer Institute University of Utah Salt Lake City, UT United States; 3 National Security Sciences Directorate Oak Ridge National Lab Knoxville, TN United States

**Keywords:** racial/ethnic stratification, geo-tagged COVID-19 tweets, racial/ethnic disparity, surveillance

## Abstract

**Background:**

The COVID-19 pandemic exacerbated existing racial/ethnic health disparities in the United States. Monitoring nationwide Twitter conversations about COVID-19 and race/ethnicity could shed light on the impact of the pandemic on racial/ethnic minorities and help address health disparities.

**Objective:**

This paper aims to examine the association between COVID-19 tweet volume and COVID-19 cases and deaths, stratified by race/ethnicity, in the early onset of the pandemic.

**Methods:**

This cross-sectional study used geotagged COVID-19 tweets from within the United States posted in April 2020 on Twitter to examine the association between tweet volume, COVID-19 surveillance data (total cases and deaths in April), and population size. The studied time frame was limited to April 2020 because April was the earliest month when COVID-19 surveillance data on racial/ethnic groups were collected. Racially/ethnically stratified tweets were extracted using racial/ethnic group–related keywords (Asian, Black, Latino, and White) from COVID-19 tweets. Racially/ethnically stratified tweets, COVID-19 cases, and COVID-19 deaths were mapped to reveal their spatial distribution patterns. An ordinary least squares (OLS) regression model was applied to each stratified dataset.

**Results:**

The racially/ethnically stratified tweet volume was associated with surveillance data. Specifically, an increase of 1 Asian tweet was correlated with 288 Asian cases (*P*<.001) and 93.4 Asian deaths (*P*<.001); an increase of 1 Black tweet was linked to 47.6 Black deaths (*P*<.001); an increase of 1 Latino tweet was linked to 719 Latino deaths (*P*<.001); and an increase of 1 White tweet was linked to 60.2 White deaths (*P*<.001).

**Conclusions:**

Using racially/ethnically stratified Twitter data as a surveillance indicator could inform epidemiologic trends to help estimate future surges of COVID-19 cases and potential future outbreaks of a pandemic among racial/ethnic groups.

## Introduction

As the novel SARS-CoV-2 virus, which causes COVID-19, started spreading worldwide in early 2020, people began practicing social distancing as a measure to reduce contagion [1]. The public also started using Twitter to exchange information about the pandemic, which contributed to the exponential increase in social media traffic volume [2]. According to the Centers for Disease Control and Prevention (CDC), as of March 31, 2022, the pandemic had claimed over 995,000 lives in the United States, which continues to rise [3,4]. This is the worst pandemic the United States has experienced since the 1918 flu [5].

Racial/ethnic disparities in health in the United States have been well documented. Stark racial disparities exist in health outcome measures, health care, and chronic health conditions [6,7]. In almost all health outcome measures, these disparities are precipitated by the disproportionate representation of Black Americans, Hispanic/Latino Americans, Asian Americans, and other racial/ethnic minorities [6,8] and often due to limited access these communities have to hospitals and health care facilities [9]. The COVID-19 crisis has further exacerbated those disparities because racial/ethnic minorities have higher representation in sectors most affected by the pandemic and are more likely to be employed in low-wage or precarious jobs [8,10,11].

An increasing number of public health and medical agencies are using Twitter data to monitor disease trends and detect outbreaks at the national and local levels [12,13] (ie, dental pain [14], cancer [13], as well as a syndromic surveillance system [15]). Twitter data, especially geotagged tweets, have several advantages over traditional surveillance data. Traditional surveillance systems, such as surveys or clinical data collection, are expensive and time-consuming monitoring mechanisms [16]. Tweets, on the other hand, are more timely and less costly [16,17]. In addition, the geographic extent of tweet data makes it easy to conduct multiscale studies, from a neighborhood to counties to across multiple nations [18]. Geotagged tweets can also be geocoded to match exact location information, making it possible to link to other data sources based on common geographical attributes (ie, state, county, or local address) for geospatial analysis [19]. By contrast, most publicly available traditional surveillance data have restricted geographic variables, and county level is often the finest spatial resolution as these data need to be aggregated from a privacy perspective [20-22].

In 2020, Twitter data alone was used for the national surveillance of COVID-19 hospitalizations in Belgium [1], COVID-19–related anti-Asian sentiments in the United States [23], evaluating world leaders’ COVID-19 response measures [24], and tracking mental health symptoms mentioned during the COVID-19 pandemic within the United States [25]. In particular, the relation between tweet volume and surveillance was explored for various purposes [26-29]. Increased tweet volume is often assumed to correlate with increased public interest in certain topics. Subsequently, this approach was used as a measure to monitor public discourse on mask-wearing [26], increased COVID-19 cases [27], and predicting outbreaks [28,29].

Despite these great benefits, Pobiruchin et al [27] pointed out the need and importance of investigating the potential correlation between tweet volume and infection or death rates; Nguyen et al [23] suggested future work on the geographical variation in area-level COVID-19 infection and mortality rates and their associations with demographics such as density of racial/ethnic groups. Considering the research gaps and the context of the COVID-19 pandemic’s disproportionate impact on racial/ethnic minorities, our goal was to explore the association between racially/ethnically stratified tweets and COVID-19 cases and deaths. We believe this study will reveal the potential relation of public discourse about the pandemic’s impact on each race/ethnicity group and COVID-19 cases and deaths. Using racially/ethnically stratified tweet volume as a surveillance indicator will provide more evidence and help address racial/ethnic health disparity in this COVID-19 crisis.

## Methods

### Data Collection

English tweets geotagged within the United States from April 1, 2020, to April 30, 2020, were downloaded using the Tweepy Python library—Twitter’s search application programming interface (API) —and a set of predefined search terms [30]. Referencing the most searched terms from Google’s Daily Search Trends summary from March 10, 2020, to April 1, 2020, we derived a list of search terms. The search terms included (1) the most widely adopted scientific name of the disease (“corona,” “COVID-19,” “pandemic,” “coronavirus”), (2) the name of the racial/ethnic groups in which we were interested (“Asian,” “Blacks,” “Hispanics,” and “Latino”), and (3) other related terms (“test positive,” “COVID,” “n95,” “Flatten the curve,” “Social Distancing,” “Chinese Virus”; see the complete list of keywords in Multimedia Appendix 1). We only included geotagged tweets with location information (ie, geographical coordinates and city names) to filter tweets within the United States. The location information allowed us to match tweets to specific states and then link state-level surveillance data for further analysis. Each tweet’s text, timestamp, coordinates, or place names were extracted and used in the detailed analysis.

COVID-19 cases were the “total number of confirmed plus probable cases of COVID-19 reported by the state or territory” for April 2020 obtained from The COVID Tracking Project [31]. COVID-19 deaths were “total fatalities with confirmed or probable COVID-19 case diagnosis” from The COVID Tracking Project, the only source with data about race across all states. These data cannot be found in neither the CDC COVID Data Tracker nor the COVID-19 Dashboard at John Hopkins University. State-level population data for each racial/ethnic group were obtained from the US Census Bureau’s 2019 American Community Survey 5-year estimates [32].

### Spatial and Statistical Analysis

**Figure 1:**
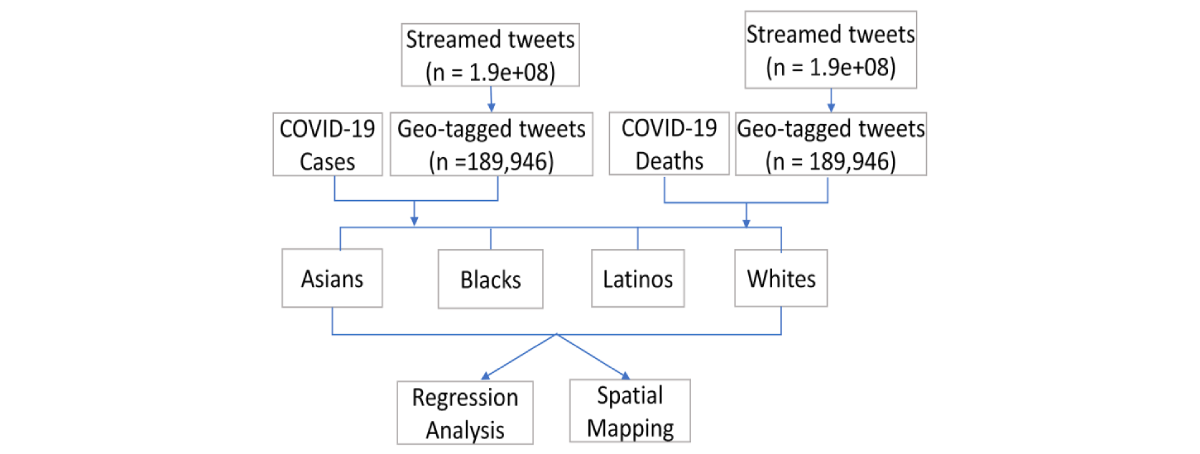
Data management and workflow.

Figure 1 illustrates the organization of the tweets and COVID-19 case and death data and the analytics implemented to examine the racial/ethnic relationships. First, the tweets were cleaned using natural language processing steps, removing punctuation, English stop words (ie, a, an, the), and special characters. Using the name of each racial/ethnic group, we extracted tweets that contained conversations about the subpopulations of interest: Asians, Blacks, Latinos, and Whites. If multiple racial/ethnic groups were mentioned in 1 tweet, the tweet was examined multiple times. “Black” and “White” can also refer to the name of colors. To exclude tweets that used “Black” and “White” but did not mean ethnic groups, the tweets were manually evaluated to identify and exclude irrelevant tweets. Next, we calculated the total number of tweets (or tweet volume) for Asians, Blacks, Latinos, and Whites in 50 states and Washington, DC, based on geotagged information.

COVID-19 case and death data were recorded twice a week, so we combined these data to yield the total cases and deaths for April 2020. To map and compare the cases and deaths across racial/ethnic groups, we used 2019 US Census population data to adjust the number of cases or deaths per 100,000 Asians/Blacks/Latinos/Whites. The population-adjusted cases and deaths were mapped using the geospatial processing tool ArcPro 2.5 (ESRI, 2020; Redland, CA) to allow comparison across racial/ethnic groups.

Using the state name as the common field, we linked tweet volume, COVID-19 cases and deaths, and population estimates for the 50 states and Washington, DC. Four ordinary least squares (OLS) regressions were implemented for Asians, Blacks, Latinos, and Whites, respectively, to examine the association of tweet volume with COVID-19 cases. In each model, tweet volume was used as the independent variable, the population was the control variable, and the number of COVID-19 cases was the outcome variable. Another 4 OLS regressions were implemented for Asians, Blacks, Latinos, and Whites, respectively, to examine the association between tweet volume and COVID-19 deaths. Tweet volume, population, and COVID-19 deaths were used as each model’s independent, control, and outcome variables, respectively.

## Results

The final analysis included 189,946 geotagged tweets. Of these, we extracted 141 (0.07%) tweets mentioning Asians, 869 (0.46%) tweets about Blacks, 47 (0.02%) tweets about Latinos, and 588 (0.31%) tweets about Whites. Example tweets about each racial/ethnic group are presented in Table 1.

**Figure 2:**
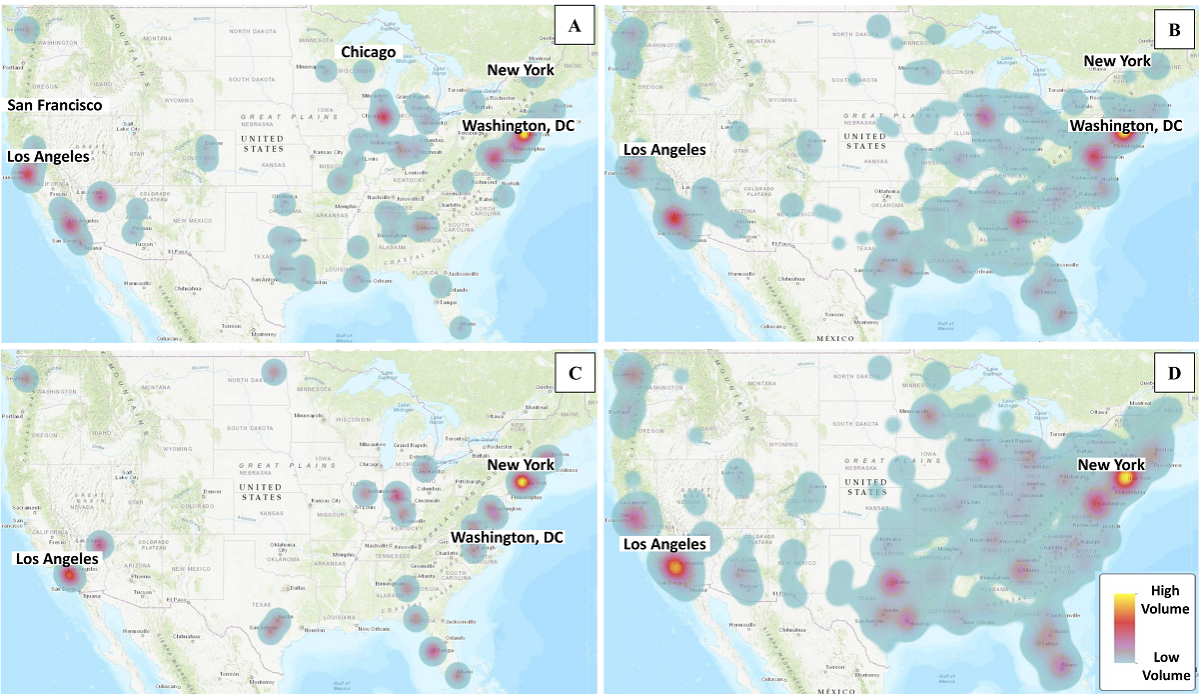
Distribution of the volume of tweets related to conversations about COVID-19 and (A) Asians, (B) Blacks, (C) Latinos, and (D) Whites.

The spatial locations of tweets were mapped, and hot spots based on tweet volume were identified (Figure 2). These hot spots (gradient from red to yellow indicates increasing volume) include New York, Los Angeles, and Chicago. The non-hot spot locations (blue-gray color) represent low tweet volume.

**Figure 3:**
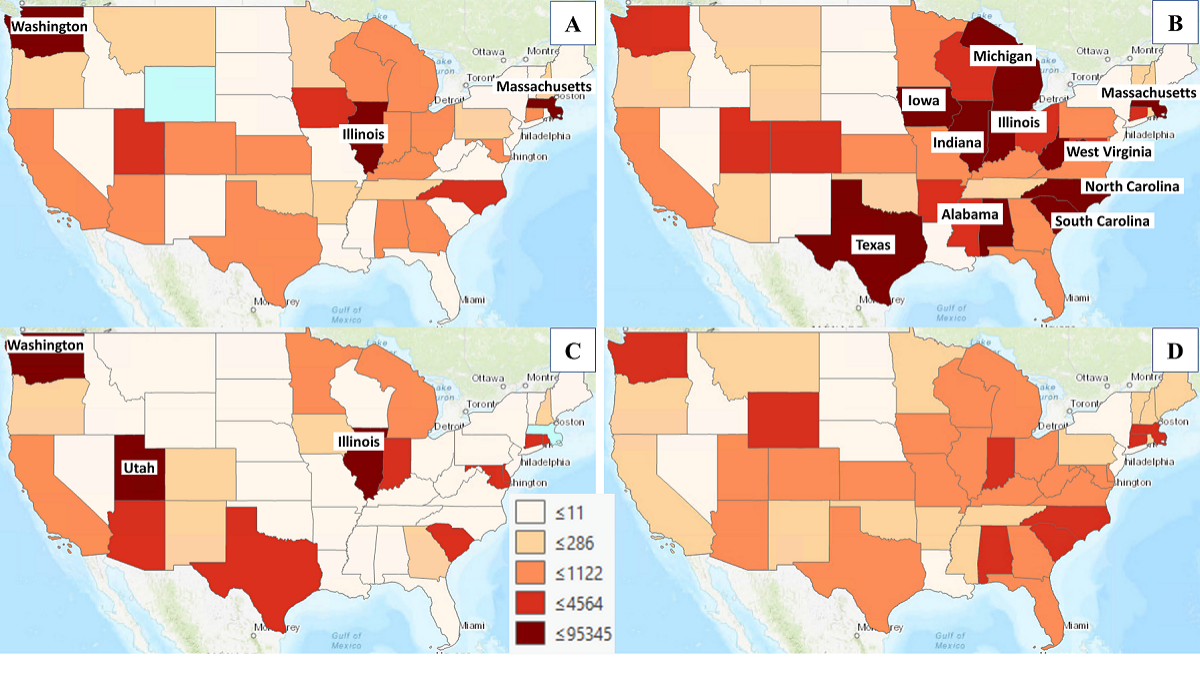
COVID-19 cases for (A) Asians (the blue for Wyoming indicates no data), (B) Blacks, (C) Latinos, and (D) Whites.

The distributions of population-adjusted COVID-19 cases for Asians, Blacks, Latinos, and Whites in April 2020 are shown in Figure 3. Alaska and Hawaii are not shown on the maps because the cases were few for Asians and close to 0 for the other 3 racial/ethnic groups in both states in April 2020. The number of COVID-19 cases (≤95,345 per 100,000) among Asians was highest in Illinois, Massachusetts, and Washington. Similarly, Latino cases were highest in Illinois, Utah, and Washington. The highest cases among Blacks were in the Midwest, Northeast, and Southern regions (eg, Illinois, Massachusetts, Alabama, among others). Compared with Asians and Blacks, the number of COVID-19 cases among Whites was the lowest across all states.

**Figure 4:**
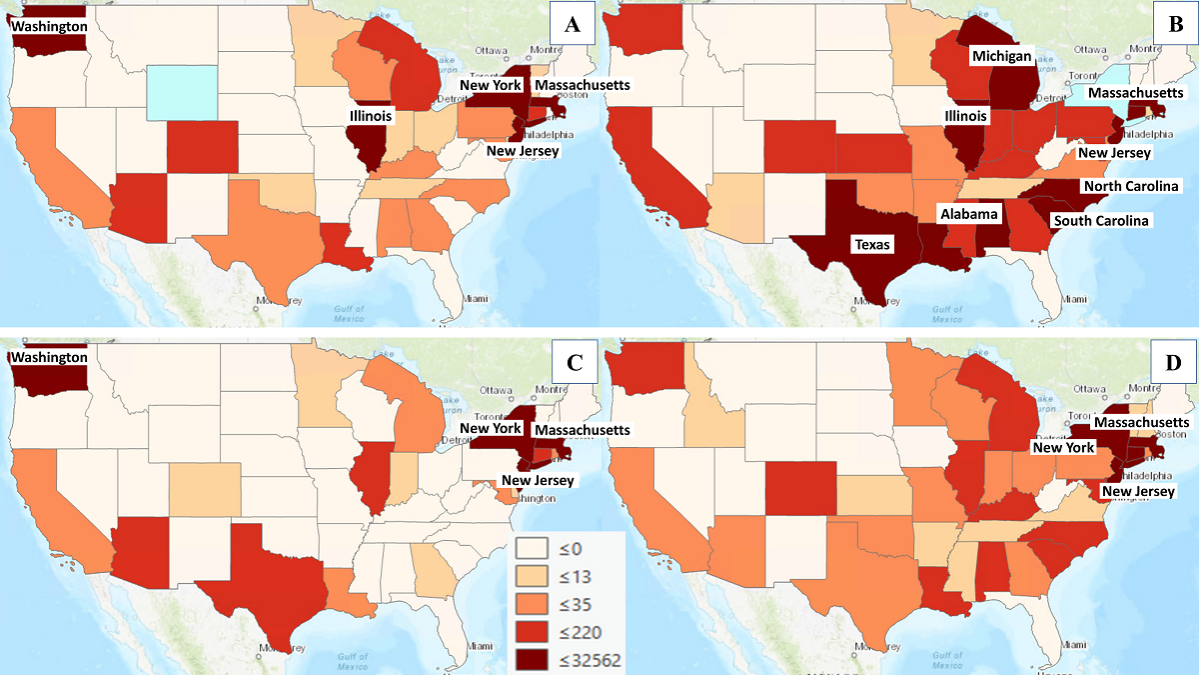
COVID-19 deaths for (A) Asians, (B) Blacks, (C) Latinos, and (D) Whites.

Figure 4 shows the distributions of population-adjusted COVID-19 deaths for Asians, Blacks, Latinos, and Whites in each state for April 2020. The number of deaths among Asians was highest (>220 per 100,000) in Illinois, Massachusetts, New York, and Washington. Massachusetts, New York, and Washington were the states that reported the highest numbers of death for Latinos (>220 per 100,000). Similar to the number of infected cases, the number of deaths among Blacks was also highest (>220 per 100,000) in the Midwest, Northeast, and Southern regions (ie, Alabama, Illinois, Massachusetts, Michigan, New Jersey, New York, North Carolina, South Carolina, and Texas).

Tables 2 and 3 show the linear regression results between COVID-19 cases and tweet volume. We observed a significant relationship between Asian tweet volume and the number of COVID-19 cases. Specifically, an increase of 1 Asian tweet was linked to 288 Asian cases (Model 1, adjusted *R*^2^=0.72, *P*<.001). The rise of Black, Latino, and White tweets was not correlated with COVID-19 cases. Furthermore, tweet volume for all racial/ethnic groups was significantly associated with COVID-19 deaths: An increase of 1 Asian tweet was linked to 93.4 Asian deaths (Model 5, adjusted *R*^2^=0.57, *P*<.001); an increase of 1 Black tweet was linked to 47.6 Black deaths (Model 6, adjusted *R*^2^=0.23, *P*<.001); an increase of 1 Latino tweet was linked to 719 Latino deaths (Model 7, adjusted *R*^2^=0.38, *P*<.001); and an increase of 1 White tweet was linked 60.2 White deaths (Model 8, adjusted *R*^2^=0.18, *P*<.001).

**Table 1:** Example tweets about each racial/ethnic group.

Ethnic group	Example tweets
Asians	“Eating at #Italianos for the #1stTime. My #Wife told Me not to eat any #AsianFood because the #Covid19 started in Asia.” “Asian fam, stay safe out there. We 'bout to be targeted much more now than ever. This is an okay time to be paranoid. sadly.” “A terror attack in Texas due to anti-Asian hatred and bigotry. THIS is why it is appalling and abhorrent to apply a nationality and ethnicity to a f*cking virus.” “Latinos and Asians in New York City are disproportionately representing the proportion of COVID-19 cases.”
Blacks	“Our COUNTRY is shut down, not because of a black guy, but because of a white guy @realDonaldTrump#COVID19.” “I am disappointed in my fellow blacks for being so ignorant during this time. I just read a comment that said “they puttin the virus in the COVID tests now that Black people being tested, so they get sick.” “There is research showing that Black women and other minorities aren’t believed when they report symptoms in the E.R. Could this lead to major racial disparities in the survival rates of COVID-19 patients?#CNNTownHall”
Latinos	“I live in one of the epicenters of the epicenter of this damn #COVID19 crisis. Working class Black & Latino folks. When will we get tested?” “Important. Even here in Iowa, #COVID19 is disproportionately impacting Black and Latino people.” “As of April 12, a total of 31 Latinos have died from complications from COVID-19, according to data from the medical examiner’s office. But that figure is not accurate.” Via @mizamudio “Younger blacks and Latinos are dying of COVID-19 at higher rates in California.”
Whites	“White nationalists looking to weaponize coronavirus pandemic, both literally and figuratively.” “Striking maps of Milwaukee by overlaying COVID cases on high African American (left) or White population.” “White supremacy backfiring on Trump. He closed the border to China, but not Europe. Most covid-19 cases in USA have a Euro/British origin.” “Same people ok with a bunch of white folks parading around with guns protesting a pandemic had a problem with black folks protesting police brutality and injustice.”

**Table 2:** Association between COVID-19 cases and racial/ethnic group–related tweets.

Model, outcome, and measurement variable		Coefficient	*P* value	Confidence interval	Adjusted *R*^2^
**Model 1: Asian COVID-19 cases**					
	Asian tweet volume	288.192	0.00	162.3 to 414.0	0.72
	Asian population	0.002	0.00	0 to 0	
**Model 2: Black COVID-19 cases**					
	Black tweet volume	97.088	0.28	–80.3 to 274.5	0.08
	Black population	0.004	0.07	0 to 0	
**Model 3: Latino COVID-19 cases**					
	Latino tweet volume	2161.828	0.05	–24.0 to 4347.7	0.26
	Latino population	0.001	0.00	0 to 0	
**Model 4: White COVID-19 cases**					
	White tweet volume	163.938	0.07	–12.3 to 340.1	0.19
	White population	0.001	0.03	0 to 0	

**Table 3:** Association between COVID-19 deaths and racial/ethnic group–related tweets.

Model, outcome, and measurement variable		Coefficient	*P* value	Confidence interval	Adjusted *R*^2^
**Model 5: Asian COVID-19 deaths**					
	Asian tweet volume	93.3750	0.00	68.9 to 117.7	0.57
	Asian population	–0.0001	0.02	0 to 0	
**Model 6: Black COVID-19 deaths**					
	Black tweet volume	47.6001	0.00	24.1 to 71.0	0.23
	Black population	–0.0002	0.39	0 to 0	
**Model 7: Latino COVID-19 deaths**					
	Latino tweet volume	719.2882	0.00	463.4 to 975.1	0.38
	Latino population	–0.0001	0.29	0 to 0	
**Model 8: White COVID-19 deaths**					
	White tweet volume	60.2326	0.00	26.1 to 94.3	0.18
	White population	–0.0002	0.07	0 to 0	

## Discussion

### Principal Findings

To our knowledge, this is one of the first studies to investigate the association between racially/ethnically (Asian, Black, Latino, and White) stratified COVID-19 tweet volume and COVID-19 surveillance data (cases and deaths). Our results revealed a relationship between tweet volume and COVID-19 cases and deaths for all racial/ethnic groups in our study during April 2020 despite varying degrees of association. The key contribution of our study is the examination of the racial and ethnic bias of the pandemic using early-onset data from social media and reported cases and deaths, which was not evident early during the pandemic. This study demonstrates the importance of timely surveillance data collection to characterize the threat and spread of infectious disease among certain racial/ethnic communities, which is critical to effectively and efficiently guide a well-coordinated and targeted public health response to reduce the spreading and adverse health impacts of a pandemic.

The concentration of high tweet volumes in several hot spot locations, including New York, Los Angeles, and other places in April, could be influenced by 2 main factors. First, the regions have a large population and densely populated urban areas and a significant number of active Twitter users. Second, the increasing number of COVID-19 cases and deaths in these regions could potentially cause the COVID-19-related tweet volume to spike. The regression analysis showed that Asian tweet volume and COVID-19 cases correlated with their population. However, this association was not observed among other racial/ethnic groups. One possible reason could be the alarming increase in discrimination and hate crimes against Asians as the pandemic surged. A similar study by Nguyen et al [23] investigating negative sentiments using tweets before and shortly after COVID-19 emergence found an abnormally higher proportion of negative tweets referencing Asians than tweets about other racial/ethnic groups. The OLS results also revealed that the Asian tweet volume and Asian population were associated with Asian deaths, whereas the volume of Black, Latino, and White COVID-19 tweets was only associated with COVID-19 deaths. These different associations with tweet volume between COVID-19 cases and deaths could partially be because of restricted testing capacities for COVID-19 cases as opposed to deaths, which were actively reported and recorded. Thus, COVID-19 cases may be underestimated, and an association with COVID tweet volumes was not detected.

The findings also suggest that Twitter data, when stratified by racial/ethnic groups, could yield novel insights in using social media to detect trends in disease occurrence and the potential impact on specific racial/ethnic groups. A previous study investigated changes in racial sentiment before and after COVID-19 emergence using race-related tweets (Asian, Black, Latino, White) [23]. The study found a higher proportion of negative tweets mentioning Asians increased by more than 68%, while those relating to other racial/ethnic minorities and Whites remained stable. The timely use of tweet data is essential to detect area-level racial sentiment [23]. One side note about this previous study is that only the proportion of tweets about each race was compared and area-level comparisons were not explored; however, our research conducted area-level analysis and explored the association between tweet volume and surveillance data. Another study investigated the association between the volume of tweets against mask wearing and daily COVID-19 cases and found a rise in negative tweet volume was strongly correlated with the number of new cases [26]. This finding concurs with ours regarding the change in tweet volume associated with COVID-19 cases and deaths, although at varying degrees for different racial/ethnic groups. Different from our research, this study did not use geotagged tweets. Thus, no geographic attributes can be used to link population density, which is also a key factor influencing tweet volume. Cuomo et al [33] investigated subnational longitudinal and geospatial trends of COVID-19 in the United States between March 3, 2020, and April 13, 2020. The authors explored the association between change in tweet volume and population-adjusted COVID-19 case increase. They also conducted geospatial hot spot analysis and included population-adjusted results and found a high proportion of rural inhabitants in some of the hot spot regions, which partially overlaps with our results, as we also found high COVID-19 cases per 100,000 people in some rural states (ie, Alabama, Iowa) for the Black population.

Although previous studies have examined the relationship between tweets and COVID-19 cases, none included COVID-19 deaths as a measure in their investigations. COVID-19 deaths are an important measure because our statistical results show that all Asian, Black, Latino, and White COVID-19 tweets were associated with COVID-19 deaths but not with COVID-19 cases. One possible explanation for this is that the reporting of COVID-19 cases was limited by testing capacity in April 2020, but this limitation influenced the reporting of COVID-19 deaths less.

Future research could focus on exploring the use of Twitter to develop streamlined tools to automatically extract, process, and analyze COVID-19 (or other public health events) and racially/ethnically related tweets to monitor tweet volume about racial/ethnic groups and COVID-19–related health disparities [34]. The state-level COVID-19 surveillance data that we used limited our geospatial analysis to the state level, despite the much finer spatial resolution of the geotagged tweets. Although state-level aggregation is essential from a privacy perspective, in the future, we will consider using surveillance data at a finer spatial resolution (eg, county) to explore the variability of infected cases and deaths among different demographic groups (based on factors such as race, gender, income, age). Using early-onset Twitter data, we demonstrated a pandemic's racial/ethnic disparity among the most populous racial/ethnic groups, which could be replicated in future public health events using social media data. In the future, we will expand this study by including surveillance data for other racial/ethnic groups if this information is available.

### Public Health Implications

This retrospective study provides evidence for the use of racially/ethnically stratified Twitter data as a surveillance indicator. Social media data can provide insights to track epidemiological trends, especially for outbreaks, epidemics, or pandemics that are novel and spread fast. Such findings can aid public health practitioners and policy makers make public health decisions using this nontraditional near-real-time data set before traditional surveillance data sets are available. The research findings also encourage health care professionals to actively engage in public discourse to present scientific and clinical evidence to help reduce racial/ethnic health disparities [23,26] and eliminate misinformation. For instance, COVID-19–induced discrimination against Asians is mainly due to misunderstanding and politicizing COVID-19 origins; health care professionals can share scientific findings of possible COVID-19 origins that might not have been widely circulated [35].

The association between tweet volume and COVID-19 cases and deaths proved that tweet volume could be used as proxy surveillance data to estimate the spread of COVID-19 cases and deaths. This association can also evaluate potential locations for future COVID-19 cases and deaths. Identifying future areas for COVID-19 cases and deaths could be used for public health responses when official surveillance is not available and complement official surveillance data when available. Since Twitter surveillance is low-cost and efficient, it can be streamlined and implemented as a long-term disease surveillance tool to ensure prompt response to future public health crises.

### Limitations

Although this study used COVID-19 cases and deaths as the 2 outcomes of this disease, we are aware of the concerns about the accuracy of cases due to limited testing capabilities (ie, lack of access to testing) and challenges in the attribution of the cause of death in the early onset of the coronavirus outbreak [36]. Considering these factors, the authors acknowledge the limitation of using the counts of COVID-19 cases and deaths as variables in our models. Similar to other studies that rely on social media data, Twitter users are not representative of the population (ie, one-third of its users are between 25 and 34 years old), thus bringing selection bias to the analysis [37,38]. The exclusion of tweets in other languages, especially Spanish, also limits our understanding of information exchange between Latinos who speak Spanish.

Another limitation of this study is the spatial granularity at the state level because the surveillance data were obtained at the state level. If surveillance data can be obtained at a finer spatial resolution, then the analysis will yield more accurate and precise results and insights into the location of COVID-19 cases and deaths. Although this is a limitation of the study, given the bias towards specific racial/ethnic groups, and in order to protect the privacy of the users, state-level aggregation is a good starting point to demonstrate the methodology implemented herein to explore the racial/ethnic aspect of a pandemic.

We only used tweets collected in April 2020 and conducted a cross-sectional analysis. We acknowledge that this analysis could offer more insights into the temporal trends of conversations about different racial/ethnic groups if the data set had a larger temporal scale. However, several issues exist with extended time frame data. First, many states did not report or minimally reported race data, as it was not a requirement to report COVID-19 cases and deaths after several months into the pandemic [31]. Second, some states intentionally adjusted or manipulated their case and death data to obscure rising infection rates and mislead the public to achieve specific political goals, such as Florida's COVID-19 dashboard incident [39-41]. Since 2020, different states have implemented varying policies about social distancing and wearing masks, which probably contributed to asymptomatic cases that are difficult to identify and probably were never reported. Due to these issues, even the CDC COVID Data Tracker and COVID-19 Dashboard at John Hopkins University do not provide data about race in all states, limiting what can be studied about geographic and longitudinal racial health disparities during this pandemic.

Past research and this study have revealed that Twitter data can be used to correlate with the increased public interest in certain emergencies, such as predicting public health outbreaks [26,27] and using Twitter as a disaster situation awareness tool [42]. Twitter data are a good indicator to assess the racial/ethnic bias of the pandemic. However, we also observed that COVID-19 and race/ethnicity-related tweets significantly decreased with extended time frame data due to the aforementioned reasons. Thus, the effect of Twitter data as a surveillance indicator to inform epidemiologic trends may attenuate with time. Several reasons may have contributed to this change. For instance, the lockdown after the COVID-19 outbreak in the United States naturally brought the nation’s attention to the pandemic, and an unusually high number of people turned to Twitter to obtain or share COVID-19–related news. The politicization of the pandemic combined with the spreading of misinformation and cyber-racism [43] as well as the increase in vaccination probably contributed to a reduction in COVID-19–related tweets as we did not retrieve a significant number of tweets about COVID and race/ethnicity in December 2021 to replicate the study for the fifth wave. The continuation of this pandemic, combined with other extreme events and the economic situation that has impacted the country since 2020, probably has desensitized the public about the disproportionate impact of COVID-19 on certain racial/ethnical groups, thereby contributing to a reduction in tweets as well. Despite these limitations, similar to Cuomo et al [33], we used the data from the first month of the pandemic to understand the racial/ethnic biases of the pandemic. Hence, public health–related studies could replicate our study to explore the racial and ethnic aspects of public health problems to aid health officials with scientific communication and appropriate response measures.

### Conclusion

This research is an effort to test the possibility of using racially/ethnically stratified Twitter data as an info surveillance indicator to estimate the impact of COVID-19 on racial/ethnic groups in the United States to inform public health crisis response efforts and racial/ethnic equity. Using COVID-19 and geotagged racial/ethnic group–related Twitter data in the United States during April 2020, we filtered conversations about racial/ethnic groups in the early onset of the COVID-19 pandemic. Using the state-level counts of COVID-19 cases and deaths stratified by racial/ethnic groups, we found a strong correlation of tweet volume with COVID-19 deaths among Asians, Blacks, Latinos, and Whites and with COVID-19 cases among Asians. These findings demonstrate that racially/ethnically stratified Twitter data, as a surveillance indicator, could inform epidemiologic trends and help estimate the future surge of COVID-19 or other public health–related cases, deaths, and potential outbreaks of mutant viruses. The observed differential impacts on racial/ethnic minorities could guide public health policies to address racial/ethnic health disparities and deploy appropriate interventions (ie, more robust COVID-19 data collection about race and tailored measures to help racial/ethnic minorities).

## Appendix

Multimedia Appendix 1 Keywords used to stream Twitter data.

## Abbreviations

API: application programming interface
CDC: Centers for Disease Control and Prevention
DOE: Department of Energy
OLS: ordinary least squares
