# Correction:The Associations Between Racially/Ethnically Stratified COVID-19 Tweets and COVID-19 Cases and Deaths: Cross-sectional Study

**DOI:** 10.2196/40365

**Published:** 2022-07-06

**Authors:** Xiaohui Liu, Bandana Kar, Francisco Alejandro Montiel Ishino, Tracy Onega, Faustine Williams

**Affiliations:** 1 National Institute on Minority Health and Health Disparities National Institutes of Health Bethesda, MD United States; 2 Huntsman Cancer Institute University of Utah Salt Lake City, UT United States; 3 National Security Sciences Directorate Oak Ridge National Lab Knoxville, TN United States

In “The Associations Between Racially/Ethnically Stratified COVID-19 Tweets and COVID-19 Cases and Deaths: Cross-sectional Study” (JMIR Form Res 2022 May 30;6(5):e30371. doi: 10.2196/30371), one error was noted.

In the original article author Faustine Williams was incorrectly associated with affiliation 2:

Faustine Williams^2^, MPH, PhD

^2^Huntsman Cancer Institute, University of Utah, Salt Lake City, UT, United States.

The correct affiliation should be affiliation 1:

Faustine Williams^1^, MPH, PhD

^1^National Institute on Minority Health and Health Disparities, National Institutes of Health, Bethesda, MD, United States.

The correction will appear in the online version of the paper on the JMIR Publications website on July 6, 2022, together with the publication of this correction notice. Because this was made after submission to PubMed, PubMed Central, and other full-text repositories, the corrected article has also been resubmitted to those repositories.

