# Supplementary material for: The Associations Between Racially/Ethnically Stratified COVID-19 Tweets and COVID-19 Cases and Deaths: Cross-sectional Study
Source: JMIR Form Res. 2022 May 30;6(5):e30371. doi: 10.2196/30371 (PMC9153911; doi:10.2196/30371)
Supplement: Multimedia Appendix 1 [file formative_v6i5e30371_app1.docx]

**Appendix 1. Keywords used to stream Twitter data**

'test positive' , 'covid' , 'covid19' , 'cronavirus' , 'confirmed cases' , 'TrumpMeltdown' , 'Fauci' , 'quarantine' , 'FLATTEN THE CURVE' , 'PanicBuying' , '2019-nCov' , 'Outbreak' , 'Virus' , 'Pandemic' , 'Infection' , 'NIAID' , 'Biogen' , 'CDC' , 'WHO' , 'Chinese American', 'Asian American', 'shutdown','covid','lockdown','Hispanic','blacks', ‘white’, 'n95', 'minority', 'layoffs', '#lifeafterlockup', '#ThankYouPresidentTrump', '#CoronaCrisis', '#CALockdown', '#Quarantine', '#SOCIALDISTANCING', '#FLATTENTHECURVE', '#CHINESEVIRUS', '#Covid19', '#Coronavirus', '#2019Ncov', '#Sarscov2', '#Wuhan', '#Covid-19', '#Covid2019', '#Covid_19', '#COVIDIOT'
